# Pain Management After Open Liver Resection: Epidural Analgesia Versus Ultrasound-Guided Erector Spinae Plane Block

**DOI:** 10.7759/cureus.28185

**Published:** 2022-08-19

**Authors:** Jesse W Stewart, Adam Yopp, Matthew R Porembka, John D Karalis, Mary Sunna, Cedar Schulz, John C Alexander, Irina Gasanova, Girish P Joshi

**Affiliations:** 1 Department of Anesthesiology and Pain Management, University of Texas Southwestern Medical Center, Dallas, USA; 2 Division of Surgical Oncology, Department of Surgery, University of Texas Southwestern Medical Center, Dallas, USA; 3 Department of Surgery, University of Texas Southwestern Medical Center, Dallas, USA; 4 Department of Nursing, Parkland Health and Hospital System, Dallas, USA

**Keywords:** erector spinae plane block, thoracic epidural anesthesia, regional analgesia, postoperative pain, liver resection

## Abstract

Background: Multimodal analgesia techniques, including regional analgesia, have been shown to provide effective analgesia and minimize opioid consumption after liver resection surgery. While thoracic epidural analgesia (TEA) is considered the gold standard, its role in the current era of enhanced recovery after surgery (ERAS) has been questioned. Erector spinae plane blocks (ESPBs) have the potential to provide effective postoperative analgesia without the risks associated with epidural analgesia. The primary aim of this quality improvement project was to evaluate the analgesic efficacy of ultrasound-guided ESPB in comparison with TEA in patients undergoing open liver resection.

Methods: Fifty patients who underwent open liver resection and received TEA (n=25) or ESPB (n=25) as part of an ERAS pathway were retrospectively identified. The primary outcome measure was cumulative postoperative opioid consumption at 24 hours. Secondary outcomes included opioid consumption, pain scores, the incidence of nausea and vomiting requiring antiemetics, lower extremity muscle weakness, and occurrence of hypotension requiring treatment on arrival to the post-anesthesia care unit and at 2, 6, 12, 24 hours, and daily through postoperative day 7.

Results: Opioid requirements were significantly lower in the TEA group compared to the ESPB group. Postoperative pain scores at rest and with deep inspiration were significantly lower in the TEA group through postoperative day 5. There were no differences in other outcome measures.

Conclusions: These findings suggest that compared with ESPB, TEA provides superior pain relief after open liver resection.

## Introduction

Adequate pain control improves postoperative outcomes and is imperative for enhanced recovery after surgery (ERAS) [1-3]. Open liver resection surgery is associated with intraoperative blood loss, hypotension, coagulopathy, pulmonary complications, liver impairment, and renal impairment, making perioperative pain management challenging [4]. Multimodal analgesic strategies employing regional techniques decrease postoperative pain and opioid consumption following liver resections [5]. Thoracic epidural analgesia (TEA) is considered the ‘gold standard’ for open thoracic and abdominal surgical procedures [1,2]. However, there are concerns regarding associated complications such as perioperative hypotension and delayed catheter removal due to potential coagulopathy, which may delay ambulation and overall recovery [1,2]. Therefore, in the era of ERAS, the role of TEA has been questioned, particularly because alternative regional analgesic techniques (e.g., interfascial plane blocks) have been reported to provide similar recovery outcomes [1,2,6].

The erector spinae plane block (ESPB) provides both visceral and somatic analgesia and has been shown to provide effective postoperative pain control over a wide spectrum of surgical procedures including thoracic, breast, abdominal, and spinal surgeries [7-11]. ESPBs are easy to perform, have a low complication rate, and are comparatively safe to perform in the presence of systemic coagulopathy [12], which is common in patients with liver disease. Recently, the Procedure-Specific Pain Management (PROSPECT) published guidelines for postoperative pain management following liver surgery; however, ESPBs were not included because of sparse evidence in patients undergoing liver resections [2]. The aim of this quality improvement project was to evaluate the analgesic efficacy of preoperative ultrasound-guided ESPB compared to TEA as part of a multimodal analgesic regimen in patients undergoing open liver resections. 

## Materials and methods

This quality improvement project was performed at Parkland Health and Hospital Systems, Dallas, Texas. A waiver of documentation for informed consent was obtained from the Institutional Review Board of the University of Texas Southwestern Medical Center, Dallas, Texas (STU-2021-1059). Patients scheduled to undergo elective liver resections under general anesthesia between January 2019 and September 2021 who received either TEA or right-sided ESPB with catheter placement as part of a multimodal opioid-sparing pain management pathway were included. Exclusion criteria included age <18 years, American Society of Anesthesiologists (ASA) physical status of 4 or greater, non-elective surgery, chronic pain conditions or chronic opioid use, contraindications to TEA or ESPB placement, coagulation abnormalities, or allergy to local anesthetic. 

The choice of epidural or ESPB placement was at the discretion of the faculty anesthesiologist and faculty surgeon. Both TEA and ESPB catheters were placed preoperatively on the day of surgery by an attending anesthesiologist. TEA placement occurred at the T7/8 interspace using a sterile technique. Subcutaneous lidocaine 1% was used at the point of entry. A 17-gauge Tuohy needle was introduced and advanced to the appropriate depth using the loss of resistance technique. The epidural catheter was advanced to a depth of 4 cm beyond the tip of the needle, and a test dose consisting of 3 mL lidocaine 1.5% and epinephrine 1:200,000 was injected to assess for intravascular placement of the catheter. The catheter was secured with adhesive wound closure strips and a clear occlusive dressing. 

Right-sided ESPB catheter placement occurred at the T7/T8 level with the patient in a sitting position. After aseptic preparation, the ultrasound transducer was placed approximately 2 cm lateral to the T7/T8 spinous processes in a parasagittal orientation. External landmarks and ultrasound imaging in real-time were used to identify the transverse process. An 18 G × 8.5 cm E-catheter (Pajunk, Germany) was introduced in-plane to the ultrasound beam, cephalad to caudad, through the paraspinous muscles until reaching the transverse process. Visualizing the catheter and needle tip under the erector spinae muscle and contacting the top of the transverse process, 20 mL of ropivacaine 0.5% was intermittently administered in 5 mL increments. The local anesthetic spread was observed in the erector spinae plane, raising the erector spinae muscle fascia off the transverse process. The needle was withdrawn, leaving the catheter in place. The catheter was then affixed to the patient’s back using a clear occlusive dressing. 

Approximately 2 hours prior to the procedure, gabapentin (600 mg, PO) was administered. Midazolam was avoided when possible, except in cases of extreme anxiety. A standardized general anesthetic technique was used for all patients as part of the liver resection surgery enhanced recovery pathway. Fentanyl (0.5-1 mcg/kg, IV, ideal body weight [IBW]), propofol (1-1.5 mg/kg, IV), and rocuronium (0.6-1 mg/kg, IV) were used for induction of general anesthesia, and a 50% mixture of oxygen/nitrous oxide and sevoflurane or desflurane (0.8-1 minimum alveolar concentration) was used for maintenance. The patient’s hemodynamics (mean arterial blood pressure and/or heart rate) were maintained within 20% of the patient’s baseline values. Fentanyl was limited to less than or about 1 mcg/kg, IV, IBW/h, excluding the induction dose. Postoperative nausea and vomiting (PONV) prophylaxis included dexamethasone 8 mg, IV, and ondansetron 4 mg, IV, administered intraoperatively. In addition, patients at high risk for PONV received a scopolamine patch prior to surgery. Hydromorphone 5-10 mcg/kg, IV, IBW was given approximately 20 minutes prior to procedure end. Upon completion of the surgery, ropivacaine 0.2% plus 2 mcg/mL of fentanyl infusion was initiated through the epidural catheter or ropivacaine 0.2% infusion through ESPB catheter and continued postoperatively at 6 mL/hour.

In the post-anesthesia care unit (PACU), patients complaining of pain received hydromorphone 0.1-0.2 mg, IV, until they were comfortable. Scheduled acetaminophen 1 g orally and cyclobenzaprine 10 mg orally were included as part of the patient’s postoperative regimen. Rescue analgesics included 0.2-0.5 mg, IV, hydromorphone every 4 hours as needed for severe pain and 5-10 mg oral oxycodone immediate release every 8 hours as needed for moderate pain. Post-operative rescue antiemetics included ondansetron 4 mg, IV, or 8 mg orally disintegrating tablet and/or 0.625 mg, IV, promethazine.

Co-authors (MS and JK) not involved in block placement extracted data from the electronic medical record, including patient demographics (i.e., age, gender, and body mass index), anesthesia and operative time, systolic and diastolic blood pressure, estimated blood loss (EBL), duration of PACU stay, time to catheter discontinuation, hospital duration, opioid prescription prior to surgery, and if the patient underwent a major hepatic resection or not. In addition, pain intensity using a 0-10 numeric rating scale (NRS) and opioid consumption (i.e., intraoperative period, PACU, and postoperative period) were recorded. Opioid doses were converted to oral morphine equivalents (OME) for normalization [13], and cumulative opioid administration was calculated for intraoperative time, time in the PACU, and at 24-hour intervals through postoperative day 7.

The primary outcome measure was cumulative postoperative opioid consumption (in OME) at 24 hours. Secondary outcomes included interval opioid consumption, NRS pain scores at rest and during deep breathing, the incidence of PONV, use of rescue antiemetics, and episodes of hypotension on arrival to the PACU at 2, 6, 12, 24 hours, and daily through postoperative day 7. Patients discharged before day 7 were contacted via telephone and their responses were recorded. Patient-reported changes in sensation and motor strength were also assessed from the medical record and recorded.

SPSS software (IBM Corp, released 2021. IBM SPSS Statistics for Windows, Version 28.0. IBM Corp, Armonk, NY) was used to perform the statistical analysis. A previous study involving the use of ultrasound-guided ESPBs for liver resection showing a mean ± standard deviation (SD) cumulative postoperative opioid consumption at 24 hours of 48.2 mg (17.1 mg) was used to calculate the sample size, with a 30% reduction in opioid consumption considered clinically significant [14]. Assuming a two-sided type 1 error of 0.05 and a power of 0.80, a required minimum sample size of 20 patients per group was calculated. To minimize any effect of data loss, more patients were included.

Continuous data were summarized as mean ± SD for normally distributed variables or as mean and 95% confidence interval (CI). Categorical data were summarized as frequency and percentages. Using normal probability plots, assumptions of normality for continuous variables were assessed. Based on the viability of normality assumption for continuous variables, Student’s t-test or Mann-Whitney U-test were used to make baseline comparisons between the two treatment groups. A repeated-measures analysis of variance (ANOVA) model with one within-subject (time) and one between-subject (treatment group) factor was used to analyze pain scores of patients at rest and during deep inspiration for each time point. This was followed up with pairwise comparisons between groups. Fisher’s exact test was used to compare incidences of nausea, vomiting, muscle weakness, sensory changes, and hypotension at each time point. Bonferroni corrections were used for multiple comparisons and statistical significance was set at p <0.05. 

## Results

Patients receiving TEA (n=25) and ESPB (n=25) were comparable with respect to patient demographics (i.e., age, sex, body mass index), duration of anesthesia, duration of PACU stay, mean blood pressure, the incidence of PONV, time to catheter removal, and time to discharge (Table 1). The number of patients who underwent a major hepatic resection was the same in both groups. The mean operative time, EBL, and presence of a prior opioid prescription were similar in the two groups (Table 1). There was no lower extremity muscle weakness reported postoperatively in either group.

**Table 1:** Patient Demographics and Clinical Variables by Treatment Group The two groups were similar with respect to patient demographics and clinical factors. Data are expressed as mean (standard deviation [SD]) or median (interquartile range [IQR]). ESPB, erector spinae plane block; TEA, thoracic epidural analgesia; PACU, post-anesthesia care unit.

	ESPB (n=25)	TEA (n=25)	p-Value
Age (years), mean (SD)	60.6 (10.2)	60.6 (10.9)	0.99
Sex (females, n (%); males, n (%))	10 (40%); 15 (60%)	10 (40%); 15 (60%)	0.99
Body mass index (kg/m^2^), mean (SD)	27.8 (5.0)	28.8 (8.9)	0.61
Systolic blood pressure (mmHg), mean (SD)	130.6 (13.4)	126.6 (49.3)	0.37
Diastolic blood pressure (mmHg), mean (SD)	77.8 (10.1)	76.36 (10.9)	0.62
Anesthesia duration (min), mean (SD)	230 (92.2)	230.1 (85.9)	0.71
Operative time (min), mean (SD)	186 (89)	193 (87)	0.78
Estimated blood loss (mL), mean (SD)	159 (169)	177 (196)	0.73
Prior opioid prescription, number of patients (%)	4 (16)	5 (20)	0.99
Major hepatic resection, number of patients (%)	8 (32)	8 (32)	0.99
PACU duration (min), mean (SD)	56.8 (46.1)	44.1 (12.9)	0.19
Time to catheter discontinued (days), median (IQR)	4 (3, 4)	3 (2.5, 4)	1.0
Time to discharge (days), median (IQR)	5 (4, 5.5)	5 (4, 6)	0.53

**Figure 1:**
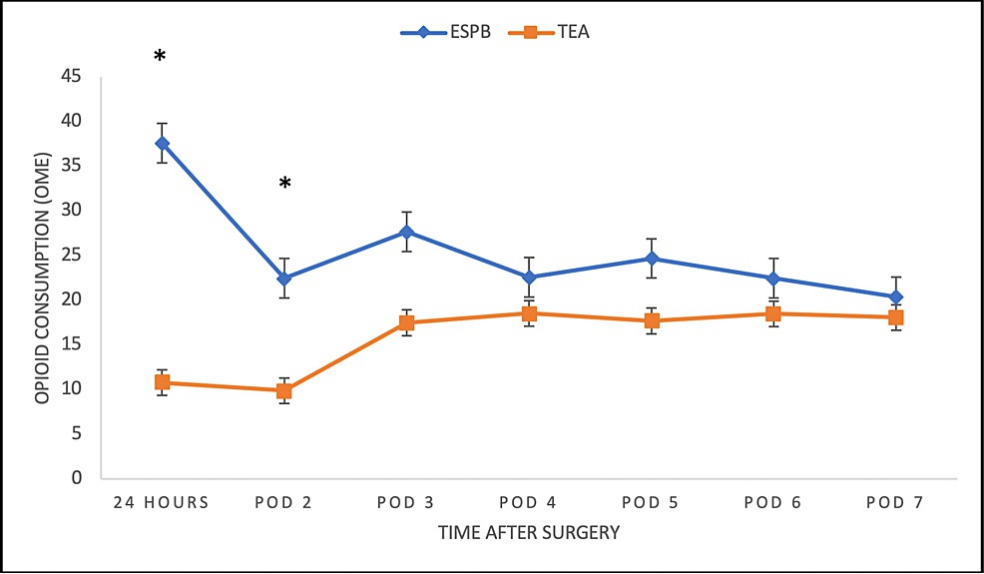
Opioid Consumption Over Time Data are expressed as mean ± standard deviation. Independent-sample t-test showed significantly lower opioid consumption for the thoracic epidural group through postoperative day 2 (p<0.002*) but no significant differences beyond postoperative day 2. ESPB, erector spinae plane block; TEA, thoracic epidural analgesia; OME, oral morphine equivalents; POD, postoperative day.

**Figure 2:**
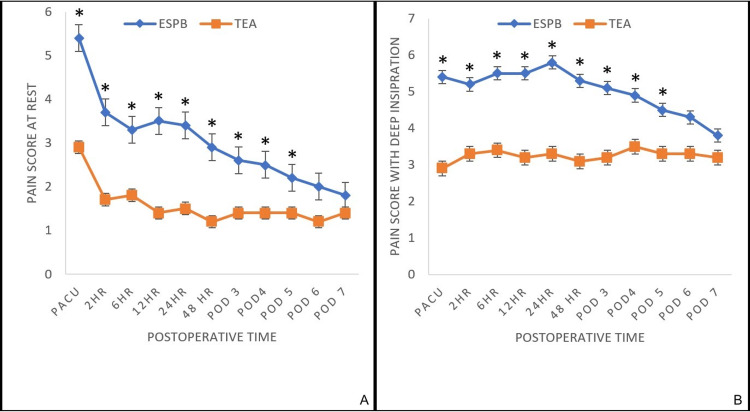
Postoperative Pain Scores at Rest and With Deep Inspiration at Various Time Points Data are expressed as mean ± standard deviation. Repeated-measures ANOVA showed a significant difference between the two groups at rest (A) and with deep inspiration (B), with the epidural group reporting lower pain scores from PACU through postoperative day 5 (p<0.001*). No statistically significant differences were detected between two groups beyond postoperative day 5. ESPB, erector spinae plane block; TEA, thoracic epidural anesthesia; PACU, post-anesthesia care unit; ANOVA, analysis of variance.

Opioid requirements in the TEA group were significantly lower for the first 24 hours (primary outcome measure) and 48 hours postoperatively (p<0.002) (Table 2, Figure 1). Overall total opioid consumption was significantly lower in the epidural group (p<0.02). Pain scores during deep inspiration and at rest for patients receiving TEA were significantly lower than those receiving ESPB through postoperative day 5 (p<0.001) (Figure 2). There were no statistically significant differences in pain scores noted between the two groups beyond postoperative day 5.

**Table 2:** Opioid Consumption Over Time by Treatment Group Opioid consumption was significantly lower in the TEA group at 24 hours and 48 hours postoperatively (*p<0.002). There were no statistically significant differences between the groups past 48 hours.  The overall total opioid consumption was significantly lower in the TEA group (†p<0.02). Data are expressed as mean (SD) or median (IQR). ESPB, erector spinae plane block; TEA, thoracic epidural analgesia; PACU, post-anesthesia care unit; SD, standard deviation; IQR, interquartile range.

	ESPB (n=25)	TEA (n=25)	p-Value
Intraoperative fentanyl (mcg), mean (SD)	303 (148.7)	306 (153.14)	0.94
PACU hydromorphone (mg), median (IQR)	0.4 (0,0.8)	0 (0, 1.3)	0.15
Morphine equivalents 0-24 h (mg), mean (SD)	37.6 (36.8)	10.8 (15.3)	0.002*
Morphine equivalents 24-48 h (mg), mean (SD)	22.5 (24.9)	9.9 (14.4)	0.002*
Morphine equivalents 48-72 h (mg), mean (SD)	27.7 (26.1)	17.5 (20.5)	0.12
Morphine equivalents Day 4 (mg), mean (SD)	22.6 (15.8)	18.56 (15.8)	0.37
Morphine equivalents Day 5 (mg), mean (SD)	24.7 (14.6)	18.7 (13.3)	0.14
Morphine equivalents Day 6 (mg), mean (SD)	22.5 (11.4)	18.5 (12.3)	0.51
Morphine equivalents Day 7 (mg), mean (SD)	20.4 (10.3)	18.1 (11.8)	0.48
Morphine equivalents total at Day 7, mean (SD)	179.9 (107.5)	113.3 (80.3)	0.02†

## Discussion

This quality improvement project demonstrated that the use of TEA was associated with significantly lower postoperative opioid consumption compared to the use of ESPB. In addition, pain scores were lower with the use of TEA. This suggests that TEA provides superior pain relief compared with ESPB. Notably, there were no differences between the groups with regard to opioid-related adverse events such as PONV or limited mobility due to lower extremity weakness. Similarly, there were no differences in clinical outcomes that could be affected by pain, such as time to ambulation.

Lower opioid use and lower pain scores with TEA may be due to greater potential for the sympathetic blockade, which may reduce visceral pain resulting during hepatectomy. Somatic and visceral pain during hepatic resection is related to a variety of factors including an incision through the skin, soft tissue, and muscle, rib retraction, as well as diaphragmatic and peritoneal irritation [5]. Somatic pain typically constitutes approximately 70-75% of pain and lasts about 72 hours following open surgery, while visceral pain is typically acute and more intense, lasting for 24-36 hours. The efficacy of ESPB in preventing visceral pain remains controversial. While Chin et al. reported the ability of ESPB to provide effective visceral analgesia in patients undergoing ventral hernia repair [15], another study reported effective somatic analgesia but less effective visceral relief [16]. Sympathetic blockade and associated hemodynamic changes (i.e., hypotension) are reflective of visceral analgesia. However, we did not observe any differences in hemodynamics (i.e., the incidence of hypotension) between the TEA and ESPB groups. This could be attributed to the current standard of practice at our institution of limiting epidural dosing until after liver resection is complete and expected blood loss is minimal. However, other measures of hemodynamic stability including intraoperative fluid use, administration of blood products, and/or vasopressor use were not evaluated, further limiting any conclusions on the hemodynamic differences between the two analgesic techniques.

While there were no differences in time to catheter removal in our study between the two groups, the potential advantages of early catheter removal in high-risk, coagulopathic or anticoagulated patients are undeniable (early ambulation, improved gut motility). ESPB catheters are considered a low-risk, easy-to-perform analgesic technique that can be safely performed in coagulopathic or anticoagulated patients [12,17,18], making it an attractive alternative to TEA when TEA placement is contraindicated or there is potential for delayed removal.

This study has several limitations. Patients were not randomized, and the determination of regional technique was left solely to the discretion of the attending anesthesiologist. Secondly, the study was not blinded, introducing a potential risk for bias. Additionally, due to ethical concerns, there was no non-regional anesthetic control group, limiting the ability to define the role of the systemic analgesic effects of local anesthesia, as well as the role of fentanyl in the TEA group. We used a right-sided catheter because the surgical incision was located in the right upper quadrant. Perhaps the use of bilateral ESPBs would have covered nerves that cross the midline. Other limitations included failure to assess the adequacy and sensory level of the block prior to surgery to preemptively identify a failed or inadequate block and the lack of clinical studies using these techniques, which limited results comparison.

## Conclusions

In summary, this quality improvement project showed an analgesic advantage of TEA over right-sided ESPB placement in patients undergoing open hepatic resection as measured by opioid consumption and pain scores at rest and on deep inspiration. While ESPB might be a safer alternative to TEA in patients at a high risk of coagulopathy, ESPB may not effectively block visceral pain associated with open liver surgery. A randomized controlled trial to assess the efficacy and safety of ESPB as a viable alternative to TEA is warranted. 

## Appendices

Enhanced recovery pathway for elective liver resection surgery

Preoperative

·   Hydrate well during the fasting period. Drink about two glasses of water before going to bed and two glasses before leaving home.

·   Pain prophylaxis:

o Gabapentin 600 mg PO 2 h preoperatively.

o Erector spinae catheter/epidural catheter for open procedures (NOT for laparoscopic approach).

§ If patient is not suitable for erector spinae/epidural analgesia, surgical site infiltration by the surgeon or four quadrant TAP blocks.

·   Scopolamine patch for very high risk postoperative nausea and vomiting (PONV).

·   Avoid midazolam, when possible.

Intraoperative

·   Prophylactic antibiotics per surgeon.

·   Bilateral SCDs.

·   General anesthetic technique (avoid deep anesthesia and limit opioid doses).

o Induction with fentanyl ~0.5-1 mcg/kg, ideal body weight (IBW), propofol (1-1.5 mg/kg, IV), and rocuronium (0.6-1 mg/kg, IV).

o Maintenance with O_2_/N_2_O 50% and desflurane or sevoflurane titrated to 0.8-1 minimum alveolar concentration (MAC).

·   Minimize intraoperative opioid dose.

o Fentanyl intraoperatively, 25-50 mcg boluses as necessary, a total of less than ~1 mcg/kg [IBW]/h (do not give morphine/hydromorphone during the procedure).

·   Ventilation: Tidal volume 6 mL/kg, positive end-expiratory pressure (PEEP) 5 cmH_2_O, respiratory rate of 8 breaths/min, initially. Maintain ETCO_2_ around 40 mmHg.

·   Maintain normothermia (core body temperature of 36-38ºC).

·   Fluid therapy: Routine central line placement for central venous pressure monitoring is not necessary.

o Initially limit fluid administration: Crystalloids less than 100 mL during induction of anesthesia and then maintenance of ~1-2 mL/kg/h).

o Administer IV infusion or sublingual nitroglycerine upon request by surgeon (to decrease portal venous pressure).

o Upon completion of resection, administer crystalloids/albumin using the goal-directed fluid therapy principles.

§ Algorithm: If baseline stroke volume variation (SVV) or systolic pressure variation (SPV) or pulse pressure variation (PPV) from arterial line >14% administer 200 mL of crystalloid, repeat until SVV or SPV or PPV <14%. Do not depend solely on SVV. Also use other parameters (e.g., urine output, hemodynamics) in conjunction with SVV.

·   Pain prophylaxis:

o Open procedures

§ Erector spinae/epidural analgesia: start infusion once majority of blood loss is over. Ropivacaine 0.2% plus 2 mcg/mL of fentanyl infusion through the epidural catheter or ropivacaine 0.2% infusion through erector spinae plane block catheter.

§ Hydromorphone 5-10 mcg/kg, IBW, at the end of surgery, approximately 20-30 minutes prior to expected time of extubation (i.e., start administration when surgical wound closure is started).

§ Open procedures with no epidural analgesia follow the protocol for laparoscopic procedures (below).

o Laparoscopic procedures: 

§ Port site infiltration with bupivacaine 0.25%, 30 mL.

§ Hydromorphone 5-10 mcg/kg, IBW or morphine 0.05-0.1 mg/kg, IBW at the end of surgery, approximately 20-30 minutes prior to expected time of extubation as noted above.

·   PONV prophylaxis with dexamethasone 8 mg after induction of anesthesia and ondansetron 4 mg at the end of surgery (if no contraindications). Ondansetron 4 mg, IV, or 8 mg oral disintegrating tablet and/or 0.625 mg, IV, promethazine as needed postoperatively.

·   Reversal of neuromuscular blockade: neostigmine dose based upon train-of-four (TOF)  response.

o TOF count 3-4-neostigmine 30-40 mcg/kg, IBW.

o TOF count 1-2-neostigmine 50-60 mcg/kg, IBW.

o No TOF response, delay reversal until at least TOF count 1.

o Ratio of neostigmine to glycopyrrolate should be 1 mg neostigmine: 0.2 mg glycopyrrolate.

·   Discontinue nasogastric tube at the end of case, if appropriate.

Postoperative

·   Pain management

o Epidural analgesia: Consider discontinuing epidural analgesia as soon as the patient can tolerate oral analgesics.

o If no epidural analgesia: IV-PCA morphine/hydromorphone. Discontinue IV-PCA as soon as the patient can tolerate oral analgesics.

o Post-anesthesia care unit.

§ Hydromorphone 0.1-0.2 mg, IV, until comfortable.

o Oral analgesia.

§ Acetaminophen 1 g, PO, and cyclobenzaprine 10 mg, PO.

§  Gabapentin until discharge: 300 mg po TID if creatinine clearance [CrCl] >60 mL/min, 300 mg PO BID if CrCl 30-60 mL/min, and 300 mg PO daily if CrCl <30 mL/min.

§ For rescue pain relief: hydromorphone 0.2-0.5 mg, IV, every 4 hours as needed for severe pain or oxycodone IR 5-10 mg, PO, as needed for moderate pain.

·   Control comorbid conditions: hypertension, blood sugar in diabetes mellitus, etc.

·   D/C urinary catheter as soon as possible, preferably postoperative day (POD) 1.

·   Fluid management: Maintenance IV fluid rate 50 mL/h lactate Ringer’s on POD 0. Bolus 250 mL lactate Ringer’s if urine output <0.5 mL/kg/h unless signs of hemodynamic instability (systolic blood pressure <90 mmHg). Wait for an hour before repeating.

·   Heplock intravenous fluid POD 1 for laparoscopic cases.

·   Nutrition: Patient encouraged to consume oral fluids (~800 mL) and liquid nutritional support ~4 h after surgery. Clear and Ensure 1 can BID after surgery postoperative day 0 soft diet on POD 1 for laparoscopic procedures and POD2 for open procedures.

·   Gum chewing encouraged.

·   Activities:

o On POD 0: Out of bed greater than 2 h, including sitting in chair and walks, if appropriate.

o Patient up in chair for all meals.

o On POD 1 until discharge: Out of bed more than 8 h including four or more walks and sitting.

Discharge Criteria

·   Surgeon discretion (in general, patient should be tolerating diet, have return of bowel function, have mobility).

·   Detailed discharge instructions, including home medications.

These recommendations are not intended to supersede clinical judgment or individual patient choices or values. Ultimately, clinical decision-making must always be customized to the individual situation.
